# *Staphylococcus aureus* Prostatic Abscess in the Setting of Prolonged *S. aureus* Bacteremia

**DOI:** 10.1155/2020/7213838

**Published:** 2020-05-20

**Authors:** Emily M. Eichenberger, Christopher J. Shoff, Robert Rolfe, Steven Pappas, Mary Townsend, Christopher J. Hostler

**Affiliations:** ^1^Division of Infectious Disease, Department of Medicine, Duke University Medical Center, Durham, NC, USA; ^2^Department of Medicine, Duke University Medical Center, Durham, NC, USA; ^3^Division of Infectious Disease, Department of Medicine, Durham Veterans Affairs Medical Center, Durham, NC, USA

## Abstract

*Staphylococcus aureus* rarely causes prostatic abscess. We report five cases of *S. aureus* prostatic abscess in the setting of bacteremia at our institution that occurred between 12/2018 and 05/2019. Three of the cases were caused by MRSA, and four of the patients underwent drainage of the prostatic abscess. All five patients received a minimum of six weeks of antibiotic therapy. One of the five patients died during the course of their infection. *S. aureus* prostatic abscess with bacteremia is an uncommon but serious disease. Treatment should consist of a combination of prolonged antibiotic therapy and surgical drainage when feasible.

## 1. Introduction

*Staphylococcus aureus* prostatic abscess is rare and incompletely understood. Prostatic abscesses are uncommon in the postantibiotic era and, when they occur, are mainly due to Gram-negative pathogens [1]. Here, we present five cases of *S. aureus* prostatic abscess with concurrent *S. aureus* bacteremia at our institution over a 6-month period from 12/2018 through 05/2019. We discuss the presentation and management of each of these patients in the context of the current literature on this topic.

## 2. Case Reports

### 2.1. Patient 1

**Figure 1:**
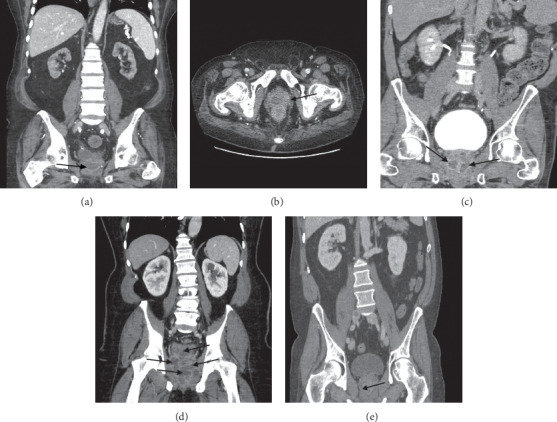
(a) Patient 1 had hypoattenuating lesions within the prostate consistent with multiple prostate abscesses; (b) patient 2 had a 2.4 cm prostatic abscess; (c) patient 3 had 2 low attenuation fluid collections within the prostate, measuring 2.6 × 1.4 cm on the right and 1.9 × 1.1 cm on the left; (d) patient 4 had a 7.8 × 7.6 × 10 cm multiloculated fluid collection with peripheral enhancement consistent with prostatic abscess; (e) patient 5 had a 2 cm prostate abscess.

A 54-year-old man with schizoaffective disorder, recurrent hepatitis C, alcohol and cocaine use, presented with suicidal ideation, weight loss, drenching night sweats, and abdominal pain over the preceding three months. A urinalysis upon admission demonstrated >100 white blood cells (WBC)/high-powered field (HPF), and 83 red blood cells (RBC)/HPF. Urine culture grew >100,000 colony forming units (CFU) of methicillin-resistant *S. aureus* (MRSA). Blood cultures were subsequently drawn and grew MRSA. His creatinine was 0.9, and his WBC was 11.9 × 10^9^/L on the day of admission. A computed tomography (CT) of the abdomen and pelvis revealed hypoattenuating lesions within the prostate consistent with multiple prostate abscesses (Figure 1(a)). A prostate exam was declined per patient request, and an indwelling urinary catheter was placed. He was initiated on intravenous (IV) vancomycin; however, after three days, he was changed to daptomycin 10 mg/kg Q24H and ceftaroline 400 mg IV Q8H due to difficulty attaining therapeutic vancomycin levels. On hospital day 7, he underwent transurethral drainage of the prostate abscesses, and the operative cultures grew MRSA. Blood cultures remained positive for 21 days. A transthoracic echocardiogram (TEE) was negative for valvular vegetations, but magnetic resonance imaging (MRI) of the spine revealed vertebral osteomyelitis. He was treated with ceftaroline and daptomycin for a total duration of 6 weeks from first sterile blood cultures, given his prolonged duration of bacteremia. This was followed by oral doxycycline, selected for prostate penetration [2], in combination with rifampin for 6 additional weeks for vertebral osteomyelitis treatment [3, 4]. A repeat CT scan performed 1 month into his anti-staphylococcal therapy demonstrated complete resolution of the prostatic abscesses.

### 2.2. Patient 2

A 69-year-old man with hypertension, hyperlipidemia, coronary artery disease, type 2 diabetes mellitus, end-stage renal disease (ESRD) on hemodialysis, and a history of toe osteomyelitis requiring prior toe amputation presented from his dialysis facility with one day of fatigue, hypotension, and fevers. He was noted to have a new diabetic foot ulcer over the dorsum of the left second toe. His WBC upon admission was 10.4 × 10^9^/L. Blood cultures from admission grew MRSA, and he was started on IV vancomycin. A TEE was negative for valvular vegetations. By hospital day 8, the therapy was changed to daptomycin 10 mg/kg and ceftaroline 200 mg IV Q8H for salvage therapy owing to persistent bacteremia [5]. He underwent CT imaging of the abdomen and pelvis, revealing a 2.4 cm prostatic abscess, a 2.6 cm right seminal vesicle abscess, and increased stranding about the bifurcation of the left common iliac artery (Figure 1(b)). A digital rectal exam revealed a smooth, nonboggy, nontender prostate without nodularity. An indwelling urinary catheter was placed, and he was immediately taken for transrectal ultrasound-guided aspiration (TRUS) of the prostatic abscess and drainage of the right seminal vesicle abscess. Cultures from the aspiration revealed MRSA in addition to several Gram-negative organisms including *Klebsiella* species and *Escherichia coli*, which were thought to be culture contaminants owing to the transrectal approach. Unfortunately, blood cultures remained positive after this procedure. A repeat CT scan was obtained five days after initial drainage and once again demonstrated the prostatic abscess and the right seminal vesicle abscess. Both appeared unchanged relative to the preoperative CT scan. It also demonstrated a soft tissue density and stranding about the bifurcation of the left common iliac artery with a new small focal outpouching of the left internal iliac artery concerning for mycotic pseudoaneurysm. He subsequently underwent left internal iliac artery coil embolization and stent graft coverage. He was discharged three days postprocedurally on ceftaroline and daptomycin with a plan to continue antibiotics for six weeks from the date of the first negative blood cultures. Notably, his blood cultures remained positive for a total of 17 days. One day after discharge, he developed the sudden onset of chest pain and suffered a cardiac arrest at home. He was brought back to the hospital and while return of spontaneous circulation was achieved in the emergency department, there were significant concerns about anoxic brain injury. The patient's family opted to pursue comfort care and he expired the following day. A postmortem examination was not performed. Blood cultures obtained during this second hospitalization were negative.

### 2.3. Patient 3

A 47-year-old man with coronary artery disease, insulin-dependent type 2 diabetes mellitus, and hyper-IgE syndrome complicated by multiple recurrent skin and soft tissue infections with MRSA, who was on suppressive doxycycline therapy, was admitted with dysuria, suprapubic abdominal pain, cloudy urine, and low-grade fevers at home. His exam was notable for suprapubic tenderness and costovertebral angle tenderness. His WBC on admission was 7 × 10^9^/L, and he had acute kidney injury with a creatinine of 1.4 mg/dL increased from his baseline creatinine of 0.8 mg/dL. A urinalysis demonstrated >100 WBC/HPF and >100 RBC/HPF. Digital rectal exam revealed a smooth, firm prostate, tender to palpation at the apex, but without bogginess or nodularity. Urine and blood cultures were obtained and rapidly grew methicillin-susceptible *S. aureus* (MSSA), which was tetracycline susceptible. He was initiated on cefazolin. CT of the abdomen and pelvis demonstrated two low attenuation fluid collections within the prostate, measuring 2.6 × 1.4 cm on the right and 1.9 × 1.1 cm on the left (Figure 1(c)). The following day he was taken for transurethral resection of the prostate abscesses and a urinary drainage catheter was left in place. Cultures from the abscess fluid grew MSSA. A TEE was then performed which was negative for valvular vegetations, and an MRI of the spine ruled out vertebral osteomyelitis or epidural abscess. Unfortunately, blood cultures drawn on hospital day 7 grew MSSA. For this reason, ertapenem was added to cefazolin based on limited in vitro and in vivo data supporting this synergistic combination [6]. The patient's blood cultures cleared by day 8 of hospitalization. After approximately one week of dual therapy with ertapenem and cefazolin, he was discharged home to complete six weeks of IV cefazolin monotherapy. His infection clinically resolved.

### 2.4. Patient 4

A 63-year-old man with hypertrophic cardiomyopathy, type 2 diabetes mellitus, hypertension, and erectile dysfunction presented with four weeks of dysuria and malaise. He first presented to his primary care physician for dysuria three weeks prior to admission at which time a urinalysis revealed pyuria and a urine culture grew MSSA. He was treated with five days of trimethoprim-sulfamethoxazole. His symptoms improved while on antibiotic therapy but recurred the following week. He subsequently presented to the emergency room wherein a second urinalysis revealed pyuria (38 WBC/HPF) and a urine culture grew MSSA. He was given another 5-day course of trimethoprim-sulfamethoxazole. His symptoms failed to improve so he returned to the emergency room ten days after his last visit with malaise, dysuria, and rectal pain. Urinalysis revealed 61 WBC/HPF, and a peripheral WBC was 17 × 10^9^/L. Digital rectal exam revealed a very large and diffusely fluctuant prostate with moderate tenderness. Blood cultures were drawn and grew MSSA. He was started on cefazolin 2 g IV Q8H. A CT scan of the abdomen and pelvis was obtained and demonstrated a 7.8 × 7.6 × 10 cm multiloculated fluid collection with peripheral enhancement consistent with a prostatic abscess (Figure 1(d)). He was taken for transurethral unroofing of the abscess the following day. Tissue cultures from the abscess grew MSSA, and his blood cultures cleared after four days. A repeat CT scan performed six days into the hospitalization showed a decreased size of the prostate abscess, measuring 5.2 × 2.1 cm, but demonstrated new central cavitary nodules in the left lung base concerning for septic emboli despite a TEE showing no valvular vegetations. He was discharged with a 6-week course of cefazolin with clinical resolution of infection.

### 2.5. Patient 5

A 55-year-old man with hypertension, hyperlipidemia, and chronic low back pain initially presented to an outside hospital for chest pain and flank pain. There, he was told he had both a urinary tract infection and pneumonia. Blood and urine cultures were collected, and he was discharged with a prescription for both cefdinir and levofloxacin but filled neither. The following day he was notified by the hospital that both his blood cultures and urine cultures grew MRSA, prompting him to present to our institution for admission. Upon arrival, a CT scan obtained showed both a 2 cm prostate abscess (Figure 1(e)) and multiple spinal epidural abscesses, which were not felt to be drainable per neurosurgery or interventional radiology. A prostate and rectal exam was deferred due to the presence of prostatic abscess on imaging. He was started on vancomycin and ceftaroline, and a urinary catheter was placed for urinary retention. Urology was consulted, but felt that given the proximity of the abscess to his sphincter muscle, he would be at high risk for permanent urinary incontinence if the abscess was drained. Blood cultures were positive for MRSA upon admission and cleared after six days of antibiotics at which time his ceftaroline was stopped. His TEE was negative for endocarditis. He was discharged with an 8-week course of vancomycin for spinal epidural abscess treatment and was subsequently transitioned to four weeks of doxycycline to complete 12 weeks in total. At the end of his 12-week course, imaging showed resolution of the prostatic abscess and spinal epidural abscesses.

## 3. Discussion

The incidence of *S. aureus* prostatic abscess is unknown. A retrospective, single-center study recently described a total of 21 cases of *S. aureus* prostatic abscess at their institution over a 10-year period, though the incidence of *S. aureus* bacteremia during this period was not reported [7]. In addition, Carroll et al. recently published a case report and comprehensive review of the literature describing a mere total of 40 cases of published *S. aureus* prostatic abscesses. They reported that MRSA was responsible for over two-thirds of the cases and that 23/40 (57.5%) of the cases were associated with concurrent bacteremia [8]. Here, we present five cases of *S. aureus* prostatic abscess during *S. aureus* bacteremia that presented within the span of six months at our institution (Table 1).

A seemingly rare phenomenon, *S. aureus* prostatic abscess, in the setting of *S. aureus* bacteremia carries substantial morbidity. Patients at increased risk for *S. aureus* prostatic abscess are immunocompromised individuals, specifically men with diabetes mellitus, chronic renal failure, or those on immunosuppressive agents [1, 8]. Intravenous drug use also appears to be a risk factor according to one study [7]. Based on our limited series and a review of the literature, we recommend that prostatic abscess should be considered in patients with complicated *S. aureus* bacteremia who present with concurrent abdominal or perineal complaints, dysuria, or urinary retention [7, 8]. It should be noted that patient 2 in our study had no genitourinary or abdominal symptoms at presentation or during his hospitalization. We believe this led to a significant delay in diagnosis of prostatic abscess and bloodstream clearance. Walker et al. similarly reported that 33% of patients with *S. aureus* prostatic abscess at their institution did not have genitourinary complaints at presentation [7]. Hence, we emphasize that patients who experience persistent *S. aureus* bacteremia despite optimal antibiotics require rigorous surveillance for persistent nidi of infection, including consideration of a prostatic abscess.

We hypothesize that all five of the patients in our cohort developed prostatic abscess as a result of hematogenous seeding of the genitourinary tract as opposed to an ascending urinary tract infection. We believe this is the case for the following three reasons: (1) none of the patients had undergone prior urethral instrumentation, (2) four of the patients had other metastatic foci of infection, and (3) the patient without an additional metastatic focus of infection had a skin/soft tissue abscess which was thought to be the source of his bacteremia. *S. aureus* is uncommonly isolated from urine cultures [9], but when present, is posited to reflect the presence of *S. aureus* bacteremia and should prompt immediate collection of blood cultures [10, 11]. Two sets of blood cultures (4 bottles: 2 aerobic and 2 anaerobic) should be sterilely and sequentially collected by venipuncture from two separate sites [12]. Blood cultures should be collected until documented clearance of *S. aureus* bacteremia [13]. Presence of *S. aureus* in one or more blood cultures is considered clinically significant, and when present should be managed according to guidelines and in consultation with an infectious disease specialist [13].

All five patients in the present series were diagnosed with prostatic abscess via CT imaging. Traditionally, the most common diagnostic modality is TRUS which is safe, radiation-free, and can be performed repeatedly to follow resolution of abscess [1, 14]. However, TRUS in a patient with a prostatic abscess may be poorly tolerated secondary to pain. Additionally, TRUS should be avoided in patients with anal fistulas, severe hemorrhoids, or abdominoperineal resection [14]. CT imaging and MRI are two alternative, suitable diagnostic modalities for prostatic abscess [14–17].

There are no guidelines for the treatment of prostatic abscess; however, we emphasize that optimal treatment consists of antimicrobials along with drainage whenever possible [18, 19]. Antimicrobial therapy for *S. aureus* bacteremia has been extensively reviewed elsewhere [13], yet data on duration of antibiotic treatment in patients with *S. aureus* prostatic abscess is unknown. All five of our patients received at least six weeks of intravenous therapy to treat for presumed *S. aureus* prostatitis [20], with two patients receiving a total of 12 weeks of therapy given the presence of concurrent MRSA vertebral osteomyelitis [21]. All three of our patients with MRSA bacteremia were treated with combination therapy involving ceftaroline. There is mounting evidence that the particular combination of ceftaroline with daptomycin is effective salvage therapy [5, 22] though the prostatic penetration of these antimicrobials has not been studied. It should be noted that prolonged combination therapy with daptomycin and ceftaroline is not the standard treatment for *S. aureus* bacteremia and was chosen in these cases as nuanced salvage therapy for persistent bacteremia.

In our cohort, four out of the five patients underwent abscess drainage. Three of the patients underwent transurethral drainage of the abscess and one underwent TRUS-guided drainage. In the comprehensive review published by Carroll et al., 26/40 (65%) patients with *S. aureus* prostatic abscess underwent drainage: 55% transurethral, 20% percutaneous, 12.5% transperineal, and 10% transrectal [8]. Indeed, there is no consensus on the optimal surgical approach for prostatic abscess drainage, though literature reviews and expert opinion suggesting that all of the above methods are effective and safe [23–26]. At our institution, surgical approach was determined on a case by case basis. We propose that early drainage of prostatic abscess in *S. aureus* bacteremia is an important means of obtaining source control as it may shorten the duration of bacteremia and reduce the risk of seeding additional foci [11, 13, 27]. Larger prospective studies are needed to identify the optimal procedure.

## 4. Conclusion

Despite its rarity, *S. aureus* prostatic abscess is associated with substantial morbidity. We recommend that prostatic abscess is considered in patients with persistent *S. aureus* bacteremia despite appropriate antimicrobial therapy. Additionally, we recommend early involvement of both an infectious disease consultant and urologist in the management of *S. aureus* prostatic abscess.

## Figures and Tables

**Table 1:** Clinical characteristics and outcomes of five patients with *S. aureus* bacteremia and prostatic abscess.

	Patient 1	Patient 2	Patient 3	Patient 4	Patient 5
Age	54	69	47	63	55
Symptoms	Weight loss, night sweats, abdomin`al pain	Fatigue, fevers	Dysuria, abdominal pain, fevers	Dysuria, malaise	Flank pain, chest pain
Comorbidities	Alcohol abuse, intravenous drug use, hepatitis C	Diabetes mellitus, coronary artery disease, end-stage renal disease on hemodialysis	Hyper IgE syndrome, diabetes mellitus, coronary artery disease	Diabetes mellitus, hypertrophic cardiomyopathy, erectile dysfunction	Hypertension, chronic low back pain
Strain	MRSA	MRSA	MSSA	MSSA	MRSA
Endocarditis	No	No	No	No	No
Metastatic foci	Vertebral osteomyelitis	Seminal vesicle abscess	None	Septic pulmonary emboli	Epidural abscess
Surgical approach	Transurethral	Transrectal	Transurethral	Transurethral	None
Days to drainage	7	8	1	1	N/A
Days to bloodstream clearance	21	17	8	4	6
Treatment	Ceftaroline and daptomycin × 6 weeks followed by rifampin and doxycycline × 6 weeks	Ceftaroline and daptomycin × 6 weeks	Cefazolin and ertapenem followed by cefazolin monotherapy × 6 weeks	Cefazolin × 6 week	Ceftaroline and vancomycin × 1 week; vancomycin × 8 weeks followed by doxycycline × 4 weeks
Outcome	Resolved	Died	Resolved	Resolved	Resolved

## Data Availability

The data used to support the findings of this study are included within the article.
